# Parâmetro que Prevê Fibrilação Atrial Pós-Operatória em Pacientes com Cirurgia de Revascularização Miocárdica: Índice de Triglicerídeos-Colesterol-Peso Corporal

**DOI:** 10.36660/abc.20240607

**Published:** 2025-03-26

**Authors:** İlhan Koyuncu, Emin Koyun

**Affiliations:** 1 Bakircay University Cigli Training and Research Hospital Department of Cardiology İzmir Turquia Department of Cardiology, Bakircay University, Cigli Training and Research Hospital, İzmir - Turquia; 2 Sivas Numune Hospital Department of Cardiology Sivas Turquia Department of Cardiology, Sivas Numune Hospital, Sivas - Turquia

**Keywords:** Fibrilação Atrial Pós-Operatória, Índice de Triglicerídeos-Colesterol-Peso Corporal, Revascularização do Miocárdio Coronário, Nutrição

## Abstract

**Fundamento:**

A fibrilação atrial pós-operatória (FAPO) é uma complicação comum após cirurgia cardíaca, particularmente cirurgia de revascularização miocárdica (CRM). Apesar dos avanços nas técnicas cirúrgicas, a FAPO continua sendo uma causa significativa de morbidade e mortalidade.

**Objetivos:**

Este estudo investiga o potencial do Índice de Triglicerídeos-Colesterol-Peso Corporal (ITCC) como um preditor de FAPO, com foco no impacto do estado nutricional nos resultados cirúrgicos.

**Métodos:**

Este estudo retrospectivo incluiu 321 pacientes submetidos à CRM entre janeiro de 2010 e janeiro de 2024. O ITCC foi calculado usando amostras de sangue pré-operatórias e comparado entre aqueles que desenvolveram FAPO e aqueles que não desenvolveram. Análises estatísticas, incluindo regressão de Cox e análise ROC, foram realizadas para avaliar o valor preditivo do ITCC para FAPO. P<0,05 foi considerado estatisticamente significativo.

**Resultados:**

Pacientes que desenvolveram FAPO apresentaram ITCC significativamente menor (1790,8 ± 689, 3413,3 ± 1232, p < 0,001, respectivamente) níveis em comparação com aqueles sem FAPO. Além disso, a idade (p < 0,001), a frequência de hipertensão (p = 0,009), PCR (p = 0,03) e valores de leucócitos (p = 0,02) também foram significativamente maiores em pacientes que desenvolveram FAPO. O ITCC foi identificado como um preditor independente de FAPO (OR: 0,998, IC de 95%: 0,997-0,999, p < 0,001), com um valor de corte de 1932,4 prevendo FAPO com sensibilidade de 75% e especificidade de 78%.

**Conclusão:**

O ITCC é um indicador confiável para prever FAPO em pacientes com CRM. A identificação pré-operatória de pacientes com baixo ITCC pode levar a intervenções direcionadas, reduzindo complicações pós-operatórias e melhorando os resultados. Otimizar o estado nutricional antes da cirurgia pode mitigar o risco de FAPO.

## Graphical abstract

**Figura Central:**
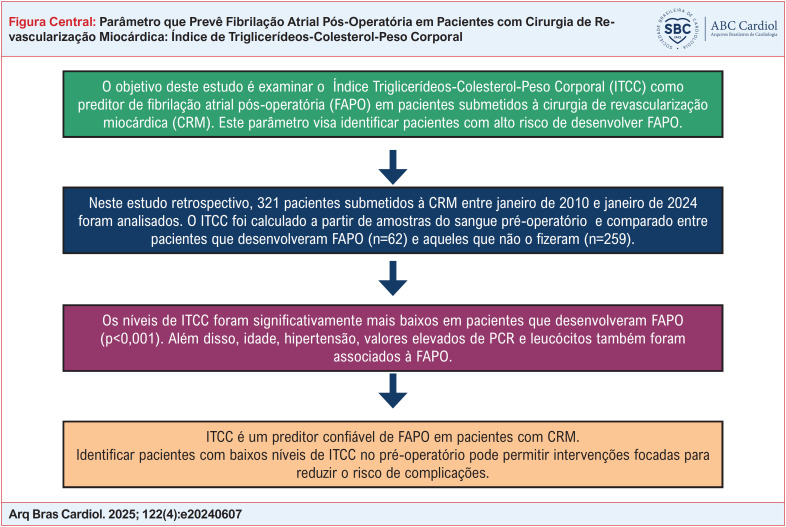
: Parâmetro que Prevê Fibrilação Atrial Pós-Operatória em Pacientes com Cirurgia de Revascularização Miocárdica: Índice de Triglicerídeos-Colesterol-Peso Corporal

## Introdução

A fibrilação atrial (FA) é uma das complicações comuns após cirurgia cardíaca e continua sendo o tipo mais prevalente de arritmia após a cirurgia. A fibrilação atrial pós-operatória (FAPO) é definida como FA que se desenvolve dentro de 1 a 5 dias após cirurgia cardíaca em pacientes sem diagnóstico prévio de FA. Felizmente, a FAPO frequentemente se converte em ritmo sinusal (RS) espontaneamente dentro das primeiras 24 horas.^1^ Estudos descobriram que a FAPO se desenvolve em 25% a 50% dos pacientes, dependendo do tipo de procedimento cirúrgico.^2^

O efeito do estado nutricional nos resultados da cirurgia cardiovascular é reconhecido há muito tempo. A nutrição inadequada aumenta o risco de complicações pós-operatórias ao melhorar processos como inflamação e estresse oxidativo. Particularmente em grandes intervenções cirúrgicas como a cirurgia de revascularização miocárdica (CRM), um bom estado nutricional no período pré-operatório pode acelerar a recuperação pós-operatória e reduzir a mortalidade.^3^ Indicadores nutricionais simples e acessíveis, como triglicerídeos, colesterol total e índice de peso corporal (ITCC), são ferramentas importantes para avaliar o estado nutricional no período pré-operatório. Baixos níveis de ITCC são considerados um sinal de desnutrição e têm se mostrado eficazes na previsão de eventos cardiovasculares em pacientes com doença arterial coronariana.^4^ Portanto, otimizar o suporte nutricional antes de grandes cirurgias como CRM é crucial para reduzir complicações pós-operatórias. A nutrição inadequada não só aumenta o risco de complicações pós-operatórias, mas também afeta negativamente a qualidade de vida geral e a sobrevida em longo prazo. Indicadores como o ITCC são ferramentas valiosas para prever esses riscos e aplicar intervenções nutricionais preventivas. Nesse contexto, avaliar com precisão o estado nutricional dos pacientes antes da cirurgia é uma etapa crítica para prevenir complicações

Apesar dos avanços nas técnicas cirúrgicas e tratamentos perioperatórios, a incidência de FAPO não diminuiu ao longo do tempo. A FAPO continua a contribuir para o aumento da morbidade e mortalidade pós-operatória.^5^ Além disso, continua sendo um fator significativo nos custos de saúde.^6^ Notavelmente, a FA é observada com mais frequência em pacientes com FAPO em comparação com aqueles em RS pós-operatório.^7^

Vários estudos demonstraram que a FAPO está associada a um aumento da incidência de complicações a curto prazo, particularmente após CRM.^8^ Além disso, estudos de longo prazo demonstraram que a FAPO está associada a um aumento do risco de morte e complicações tromboembólicas.^9^

Nos últimos anos, a pesquisa tem se concentrado em marcadores bioquímicos e metabólicos que podem prever FAPO, especialmente porque estratégias preventivas podem mitigar riscos associados. Um desses marcadores é o ITCC, calculado como:


$$\text{ITCC}=\frac{\text{Triglicerídeos (mg/dL)}\times \text{Colesterol total(mg/dL)}\times \text{Peso corporal(kg)}}{1.000}$$

Estudos anteriores mostraram que o ITCC é um marcador crítico em pacientes com insuficiência cardíaca,^10^ doença arterial coronariana,^4^ doença crítica,^11^ bem como na população em geral e pacientes com AVC.^12,13^ No entanto, a relação entre ITCC e FA, particularmente FAPO, permanece inexplorada. O estado nutricional inadequado não só aumenta o risco de complicações pós-operatórias, mas também afeta negativamente a qualidade de vida geral e a sobrevida em longo prazo. Indicadores como o ITCC são ferramentas valiosas para prever esses riscos e aplicar intervenções nutricionais preventivas. Portanto, avaliar com precisão o estado nutricional dos pacientes antes da cirurgia é uma etapa crítica na prevenção de complicações.

## Objetivo do estudo

Este estudo tem como objetivo investigar a relação entre ITCC e FAPO calculando ITCC a partir de amostras de sangue coletadas antes da CRM. Se uma associação significativa for encontrada, pode ser possível identificar pacientes de alto risco no pré-operatório e monitorá-los de perto após a cirurgia. Isso pode levar à redução da morbidade e mortalidade em pacientes propensos a desenvolver FAPO, oferecendo um novo caminho para cuidados preventivos com base em fatores de risco nutricionais e metabólicos.

## Métodos

### População de pacientes e definição de ITCC

Nosso estudo foi desenhado retrospectivamente. Entre janeiro de 2010 e janeiro de 2024, 321 pacientes consecutivos que passaram por CRM em nosso centro foram incluídos no estudo. O desenho do estudo e o fluxograma são mostrados na Ilustração Central. Os prontuários dos pacientes foram avaliados retrospectivamente. Os eletrocardiogramas (ECG) dos pacientes incluídos no estudo, feitos antes da CRM, estavam em RS. Os pacientes foram divididos em dois grupos: aqueles que desenvolveram FA pós-operatória e aqueles que não desenvolveram. Características demográficas, ITCC e parâmetros sanguíneos de ambos os grupos foram comparados. O ITCC foi calculado com esta fórmula: ‘Triglicerídeos x Colesterol total x Peso corporal/1.000’. O comitê de ética local aprovou o presente estudo. Nosso estudo foi realizado em conformidade com as diretrizes éticas da Declaração de Helsinque.

Critérios para inclusão no estudo: foram definidos como não ter diagnóstico prévio de FA, ter sido submetido a cirurgia de revascularização do miocárdio e ter um ECG sinusal documentado antes da cirurgia.

Critérios de exclusão: pacientes com distúrbios eletrolíticos, pacientes com doenças valvares cardíacas graves e insuficiência renal crônica, pacientes com marcapassos, pacientes em uso de medicamentos antiarrítmicos, pacientes com distúrbios metabólicos e pacientes sem ECG de RS pré-operatório.

A definição de FAPO foi feita da seguinte forma: em 5 dias após a cirurgia cardíaca, a arritmia com duração superior a 10 minutos resolveu-se espontaneamente ou foi tratada com cardioversão elétrica/médica.^14^ Os pacientes foram monitorados de perto para arritmia durante toda a sua estadia no hospital. Um ECG também foi feito quando ocorreram sintomas cardíacos, como palpitações. Os ECGs dos pacientes foram registrados em 12 derivações nas configurações de 10 mm/mVe 25 mm/s.

### Análise estatística

Histograma, gráfico q-q e teste de Shapiro-Wilk foram usados para avaliar se os dados violavam as suposições de normalidade. Um teste-T de duas amostras foi realizado para comparar variáveis contínuas entre os grupos. A análise qui-quadrado foi usada para avaliar a relação entre variáveis categóricas. Os dados contínuos foram apresentados como média ± desvio padrão (DP) com base na distribuição dos dados. As variáveis categóricas foram expressas como o número (n) com uma porcentagem (%). A análise de regressão de Cox foi usada para determinar os fatores de risco que afetam o estado de FAPO. As variáveis que foram consideradas estatisticamente significativas como resultado da análise de regressão de Cox foram avaliadas com análise de regressão de Cox univariada e multivariada. A análise ROC (Receiver Operating Characteristic) foi realizada para avaliar o índice ITCC e a idade na predição de FAPO. A área sob a curva e o valor de corte foram calculados para cada valor de parâmetro. A sensibilidade e a especificidade foram calculadas para determinar o poder diagnóstico dos escores. Foi aceito que os valores-p deveriam ser < 0,05 para que os parâmetros fossem estatisticamente significativos. A análise dos dados foi realizada no software estatístico SPSS 22.

## Resultados

Entre os 321 pacientes incluídos no estudo, o número de pacientes que desenvolveram FAPO após CRM foi de 62. Quando as características clínicas e demográficas basais de ambos os grupos foram comparadas, a idade dos pacientes no grupo FAPO era maior do que no outro grupo. O número de hipertensão também foi maior no grupo FAPO do que no outro grupo. O valor de proteína C-reativa (PCR) também foi maior no grupo FAPO em comparação com o outro grupo. O valor de GB também foi maior no grupo FAPO. Além disso, o ITCC foi menor no grupo FAPO do que no outro grupo. Não houve diferença significativa entre os grupos, exceto em idade, hipertensão, PCR, GB e ITCC (Tabela 1).

**Tabela 1:** Comparação das características clínicas, demográficas e laboratoriais basais entre os grupos

	Grupo FAPO (-) (n = 259)	Grupo FAPO (+) (n = 62)	Valor p
Idade, anos	61,96 ± 9,3	67,09 ± 6,5	<0,001
Gênero feminino, n (%)	65 (25,1)	16 (25,8)	0,08
Altura (cm)	170±7,1	169,3±7,3	0,72
Peso corporal (kg)	75,2±11,1	77,6±10,6	0,45
IMC (kg/m^2^)	26,80 ± 3,25	27,41 ± 3,36	0,67
Hipertensão, n (%)	190 (73,3)	55 (88)	0,009
Diabetes Mellitus, n (%)	120 (46,3)	26 (41,9)	0,35
Hiperlipidemia, n (%)	230 (88,8)	55 (88,7)	0,81
Tabagismo, n (%)	121 (46,7)	22 (35,4)	0,08
PA sistólica (mmHg)	136,6 ± 12,8	139,1 ± 16,5	0,10
PA diastólica (mmHg)	78,1 ± 8,3	79,3 ± 7,5	0,25
Creatinina (mg/dl)	0,9 ± 0,1	0,8 ± 0,15	0,17
Na (mmol/L)	138,7 ± 3,1	137,1 ± 2,2	0,90
K (mmol/L)	4,2 ± 0,2	4,3 ± 0,4	0,30
AST (U/L)	34 ±5,8	36±7,3	0,50
ALT (U/L)	31 ±6,6	36±9,2	0,51
Colesterol total	234,3 ± 14,5	221,5 ± 15,1	0,60
LDL-C	146,5 ± 9,2	159,1 ± 9,6	0,12
PCR	62,6 ± 34,3	74 ± 38	0,03
Leucócitos (103/µL)	10,08 ± 3,5	11,7 ±4,7	0,02
Neutrófilo (103/uL)	8,6±0,9	8,9 ±1,3	0,45
Hemoglobina (g/dL)	10,5 ± 1,2	11 ± 1,3	0,60
Plaquetas (103/uL)	311,4 ± 37	369,1 ± 38	0,25
FEVE (%)	53,4 ± 7,6	51,5 ± 8,1	0,18
Diâmetro do átrio esquerdo (mm)	44,2 ± 4,1	47,2 ± 5,2	0,09
LVSWT (mm)	10,3 ± 1,3	9,9 ± 2,1	0,51
Peso bruto (mm)	9,2 ± 1,5	9,6 ± 1,8	0,89
DVEDF (mm)	48,9 ± 4,1	51,1 ± 3,9	0,13
DSVVE (mm)	33,9 ± 3,4	33,4 ± 3,5	0,57
ITCC	3413,3±1232	1790,8 ± 689	<0,001

FAPO: fibrilação atrial pós-operatória, IMC: índice de massa corporal, Na: sódio, K: potássio, AST: aspartato aminotransferase, ALT: alanina aminotransferase, LDL-C: colesterol de lipoproteína de baixa densidade, PCR: proteína C-reativa, GB: glóbulo branco, FEVE: fração de ejeção do ventrículo esquerdo, LVSWT: espessura da parede septal do ventrículo esquerdo, PWT: espessura da parede posterior, LVEDD: diâmetro diastólico final do ventrículo esquerdo, LVESD: diâmetro sistólico final do ventrículo esquerdo, ITCC: triglicerídeos, colesterol total e índice de peso corporal.

Análise de regressão de Cox univariada e multivariada foi realizada para identificar variáveis independentes que predizem FAPO. De acordo com análises de regressão de Cox múltiplas, idade e ITCC foram determinados como fortes preditores independentes de FAPO após a CRM (Tabela 2).

**Tabela 2:** Análise de regressão de Cox univariada e multivariada para prever FAPO

	Univariada		Multivariada	
	Odds Ratio (IC 95%)	Valor p	Odds Ratio (IC 95%)	Valor p
Idade	1.060 (1.029 – 1.091)	<0,001	1.046 (1.014-1.080)	0,005
ITCC	0,997 (0,996 – 0,999)	<0,001	0,998 (0,997-0,999)	<0,001
Hipertensão	2.599 (1.241 – 5.445)	0,011		
PCR	1.007 (1.001 – 1.014)	0,024		
GB	1.091 (1.031 – 1.154)	0,003		

FAPO: fibrilação atrial pós-operatória; ITCC: triglicerídeos, colesterol total e índice de peso corporal; PCR: proteína C-reativa; GB: glóbulos brancos.

**Figura 1:**
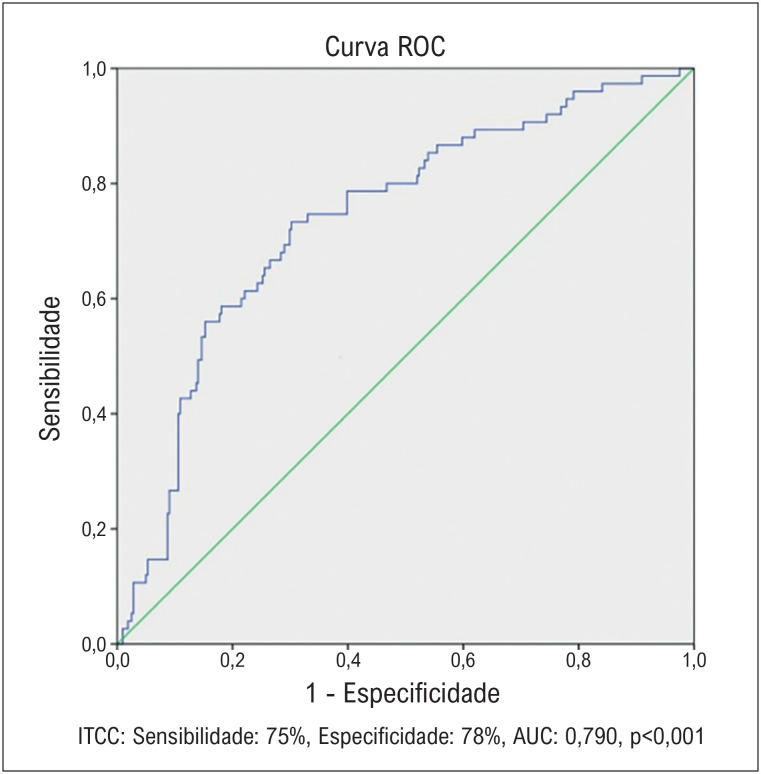
Curva ROC do Índice de Triglicerídeos-Colesterol-Peso Corporal (ITCC).

Na análise ROC, ITCC < 1932,4 foi considerado um preditor de FAPO com sensibilidade de 75% e especificidade de 78% (Figura 1).

## Discussão

Este estudo abrangente investiga a relação entre CRM e FAPO. O achado mais significativo do nosso estudo é que o ITCC foi identificado como um preditor independente de FAPO.

Historicamente, a FA não era considerada uma complicação importante após cirurgia cardíaca. No entanto, estudos subsequentes demonstraram que a FAPO impacta significativamente tanto a mortalidade quanto a morbidade.^15^ Por exemplo, um estudo conduzido com pacientes com CRM revelou que aqueles que desenvolveram FAPO experimentaram durações mais longas em ventilação mecânica, bem como estadias prolongadas na unidade de terapia intensiva e no hospital.^16^ Além disso, a FAPO foi associada a um risco aumentado de mortalidade e acidente vascular cerebral em longo prazo.^17^

Em nosso estudo, se observou que pacientes com baixos níveis de ITCC apresentaram maior risco de FAPO no período pós-operatório. Na literatura, o impacto do estado nutricional nos resultados cirúrgicos tem sido frequentemente enfatizado, e tem sido demonstrado que a desnutrição retarda o processo de recuperação e aumenta o risco de complicações.^18^ Dado o papel da inflamação no desenvolvimento da FA, acredita-se que o mau estado nutricional pode exacerbar esse processo. Foi demonstrado que a desnutrição aumenta eventos cardiovasculares e complicações após cirurgia cardiovascular.^19^ O ITCC não apenas prevê complicações como FAPO, mas também serve como um indicador sensível do estado nutricional. Essa descoberta sugere que pacientes com mau estado nutricional podem exigir suporte nutricional mais intensivo durante o período pré-operatório. Além disso, nosso estudo confirma que, em comparação com outros indicadores nutricionais, o ITCC reflete mais especificamente a desnutrição e o risco de complicações. Uma melhor compreensão da relação entre o estado nutricional e a FAPO pode contribuir para o desenvolvimento de abordagens nutricionais personalizadas nesses pacientes, reduzindo potencialmente as complicações pós-operatórias e melhorando os resultados gerais de saúde. Melhorar o estado nutricional também pode aliviar a carga sobre os sistemas de saúde, reduzindo as complicações pós-operatórias a longo prazo.

Dado o potencial da FAPO levar a complicações pós-operatórias sérias, identificar pacientes com alto risco de FA tornou-se uma meta crítica. Esse entendimento levou os pesquisadores a conduzirem estudos mais abrangentes sobre os fatores de risco associados à FAPO. É bem sabido que o mau estado nutricional desencadeia inflamação, o que pode abrir caminho para complicações como FA após a cirurgia. Índices nutricionais como o ITCC são considerados ferramentas importantes na previsão dessas complicações, e intervenções nutricionais preventivas podem ser aplicadas, particularmente em pacientes de alto risco.

Vários estudos destacaram fatores que podem contribuir para o desenvolvimento de FAPO. A hipoxemia foi identificada como um fator de risco significativo para FAPO,^20^ enquanto outras pesquisas demonstraram uma correlação entre várias técnicas cirúrgicas e a incidência de FAPO.^21^ Fatores de risco como idade avançada, aumento do diâmetro do átrio esquerdo, fração de ejeção (FE) reduzida, doença pulmonar obstrutiva crônica (DPOC), hipertensão, infarto do miocárdio e diabetes foram todos associados ao desenvolvimento de FAPO, conforme apoiado por uma meta-análise envolvendo 36.834 pacientes.^22^

O valor do ITCC como um preditor significativo de resultados em várias doenças, particularmente condições cardiovasculares, tem sido cada vez mais reconhecido. Pesquisas têm mostrado que baixos níveis de ITCC estão fortemente associados à mortalidade por todas as causas e mortalidade cardiovascular em pacientes com doença arterial coronariana.^4^ Além disso, o ITCC é recomendado para avaliar o estado nutricional de pacientes cardiovasculares, pois provou ser um forte preditor de mau prognóstico e mortalidade em pacientes cardiovasculares e na população em geral.^4^ Em nosso estudo, o ITCC foi considerado um preditor significativo de FA pós-operatória em pacientes submetidos à CRM.

Os marcadores inflamatórios também desempenham um papel significativo no desenvolvimento da FA. Muitos estudos mostraram que pacientes com FA tendem a ter níveis mais altos de marcadores inflamatórios em comparação com aqueles em RS.^23^ Além disso, os níveis de PCR demonstraram prever o desenvolvimento de FA de início recente.^24^ Em nosso estudo, contagens elevadas de glóbulos brancos (GB) e níveis de PCR no grupo que desenvolveu FAPO sugerem que um mecanismo inflamatório pode estar contribuindo para o início da FA.

A idade tem sido consistentemente identificada como um fator de risco independente para o desenvolvimento de FA. Um estudo estimou que a prevalência de FA em pacientes idosos na União Europeia mais que dobraria após os 50 anos.^25^ Nossas descobertas se alinham com essas observações, pois identificamos a idade avançada como um dos fatores de risco mais significativos para o desenvolvimento de FAPO.

### Limitações

A limitação mais importante do estudo é que ele é retrospectivo. A falta de conhecimento suficiente sobre as técnicas cirúrgicas aplicadas também é uma limitação importante. Também não temos dados sobre os medicamentos anestésicos administrados aos pacientes antes da cirurgia. Os agentes anestésicos administrados também podem ter alterado esse parâmetro. Estudos multicêntricos, prospectivos, randomizados e controlados são necessários para entender melhor se esse parâmetro é preditivo de FAPO.

## Conclusões

O ITCC pode prever o desenvolvimento de FAPO em pacientes submetidos à CRM. Com a ajuda deste parâmetro, os pacientes que precisam receber tratamento profilático antes da CRM podem ser determinados, reduzindo assim a mortalidade e a morbidade.

## References

[1] Dobrev D, Aguilar M, Heijman J, Guichard JB, Nattel S (2019). Postoperative Atrial Fibrillation: Mechanisms, Manifestations And Management. Nat Rev Cardiol.

[2] Burrage PS, Low YH, Campbell NG, O'Brien B (2019). New-Onset Atrial Fibrillation in Adult Patients after Cardiac Surgery. Curr Anesthesiol Rep.

[3] Chermesh I, Hajos J, Mashiach T, Bozhko M, Shani L, Nir RR (2014). Malnutrition in Cardiac Surgery: Food for Thought. Eur J Prev Cardiol.

[4] Maruyama S, Ebisawa S, Miura T, Yui H, Kashiwagi D, Nagae A (2021). Impact of Nutritional İndex on Long-Term Outcomes of Elderly Patients with Coronary Artery Disease: Sub-Analysis of the SHINANO 5 Year Registry. Heart Vessels.

[5] Gudbjartsson T, Helgadottir S, Sigurdsson MI, Taha A, Jeppsson A, Christensen TD (2020). New-Onset Postoperative Atrial Fibrillation after Heart Surgery. Acta Anaesthesiol Scand.

[6] Musa AF, Dillon J, Taib MEM, Yunus AM, Sanusi AR, Nordin MN (2022). Incidence and Outcomes of Postoperative Atrial Fibrillation after Coronary Artery Bypass Grafting of a Randomized Controlled Trial: A Blinded End-of-Cycle Analysis. Rev Cardiovasc Med.

[7] Ahlsson A, Fengsrud E, Bodin L, Englund A (2010). Postoperative Atrial Fibrillation in Patients Undergoing Aortocoronary Bypass Surgery Carries an Eightfold Risk of Future Atrial Fibrillation and a Doubled Cardiovascular Mortality. Eur J Cardiothorac Surg.

[8] Oraii A, Masoudkabir F, Pashang M, Jalali A, Sadeghian S, Mortazavi SH (2022). Effect of Postoperative Atrial Fibrillation on Early and Mid-Term Outcomes of Coronary Artery Bypass Graft Surgery. Eur J Cardiothorac Surg.

[9] Lee SH, Kang DR, Uhm JS, Shim J, Sung JH, Kim JY (2014). New-Onset Atrial Fibrillation Predicts Long-Term Newly Developed Atrial Fibrillation after Coronary Artery Bypass Graft. Am Heart J.

[10] Ishiwata S, Yatsu S, Kasai T, Sato A, Matsumoto H, Shitara J (2020). Prognostic Effect of a Novel Simply Calculated Nutritional Index in Acute Decompensated Heart Failure. Nutrients.

[11] Minami-Takano A, Iwata H, Miyosawa K, Kubota K, Kimura A, Osawa S (2019). A Novel Nutritional Index Serves as A Useful Prognostic Indicator in Cardiac Critical Patients Requiring Mechanical Circulatory Support. Nutrients.

[12] Fan H, Huang Y, Zhang H, Feng X, Yuan Z, Zhou J (2022). Association of Four Nutritional Scores With All-Cause and Cardiovascular Mortality in the General Population. Front Nutr.

[13] Shi Y, Wang X, Yu C, Zhou W, Wang T, Zhu L (2023). Association of a Novel Nutritional İndex with Stroke in Chinese Population with Hypertension: Insight from the China H-type Hypertension Registry Study. Front Nutr.

[14] Wong JK, Lobato RL, Pinesett A, Maxwell BG, Mora-Mangano CT, Perez MV (2014). P-Wave Characteristics on Routine Preoperative Electrocardiogram İmprove Prediction of New-Onset Postoperative Atrial Fibrillation in Cardiac Surgery. J Cardiothorac Vasc Anesth.

[15] Stamou SC, Dangas G, Hill PC, Pfister AJ, Dullum MK, Boyce SW (2000). Atrial Fibrillation after Beating Heart Surgery. Am J Cardiol.

[16] Ghurram A, Krishna N, Bhaskaran R, Kumaraswamy N, Jayant A, Varma PK (2020). Patients Who Develop Post-Operative Atrial Fibrillation have Reduced Survival after Off-Pump Coronary Artery Bypass Grafting. Indian J Thorac Cardiovasc Surg.

[17] Kosmidou I, Stone GW (2018). New-Onset Atrial Fibrillation after PCI and CABG for Left Main Disease: İnsights from the EXCEL Trial and Additional Studies. Curr Opin Cardiol.

[18] Lopez-Delgado JC, Muñoz-Del Rio G, Flordelís-Lasierra JL, Putzu A (2019). Nutrition in Adult Cardiac Surgery: Preoperative Evaluation, Management in the Postoperative Period, and Clinical Implications for Outcomes. J Cardiothorac Vasc Anesth.

[19] Yildiz I, Bayir H (2015). Malnutrition and Adverse Effects in Cardiac Surgery. Thorac Cardiovasc Surg.

[20] Wahr JA, Parks R, Boisvert D, Comunale M, Fabian J, Ramsay J (1999). Preoperative Serum Potassium Levels and Perioperative Outcomes in Cardiac Surgery Patients. Multicenter Study of Perioperative Ischemia Research Group. JAMA.

[21] Zaman AG, Archbold RA, Helft G, Paul EA, Curzen NP, Mills PG (2000). Atrial Fibrillation after Coronary Artery Bypass Surgery: A Model for Preoperative Risk Stratification. Circulation.

[22] Yamashita K, Hu N, Ranjan R, Selzman CH, Dosdall DJ (2019). Clinical Risk Factors for Postoperative Atrial Fibrillation among Patients after Cardiac Surgery. Thorac Cardiovasc Surg.

[23] Smit MD, Maass AH, De Jong AM, Kobold ACM, Van Veldhuisen DJ, Van Gelder IC (2012). Role of İnflammation in Early Atrial Fibrillation Recurrence. Europace.

[24] Aviles RJ, Martin DO, Apperson-Hansen C, Houghtaling PL, Rautaharju P, Kronmal RA (2003). Inflammation as a Risk Factor for Atrial Fibrillation. Circulation.

[25] Zathar Z, Karunatilleke A, Fawzy AM, Lip GYH (2019). Atrial Fibrillation in Older People: Concepts and Controversies. Front Med.

