# Supplementary material for: Parâmetro que Prevê Fibrilação Atrial Pós-Operatória em Pacientes com Cirurgia de Revascularização Miocárdica: Índice de Triglicerídeos-Colesterol-Peso Corporal
Source: Arq Bras Cardiol. 2025 Mar 26;122(4):e20240607. [Article in Portuguese] doi: 10.36660/abc.20240607 (PMC12040393; doi:10.36660/abc.20240607)
Supplement: Translation

# Translation: Parameter Predicting Postoperative Atrial Fibrillation in Coronary Artery Bypass Grafting Patients: Triglyceride-Cholesterol-Body Weight Index

**DOI:** 10.36660/abc.20240607i

**Authors:** İlhan Koyuncu, Emin Koyun

**Affiliations:** 1 İzmir Türkiye Department of Cardiology, Bakircay University, Cigli Training and Research Hospital, İzmir - Türkiye; 2 Sivas Türkiye Department of Cardiology, Sivas Numune Hospital, Sivas - Türkiye

**Keywords:** Postoperative Atrial Fibrillation, Triglyceride-Cholesterol-Body Weight Index, Coronary Artery Bypass Grafting, Nutrition

## Abstract

**Background:**

Postoperative atrial fibrillation (POAF) is a common complication after cardiac surgery, particularly coronary artery bypass grafting (CABG). Despite advances in surgical techniques, POAF remains a significant cause of morbidity and mortality.

**Objectives:**

This study investigates the potential of the Triglyceride-Cholesterol-Body weight Index (TCBI) as a predictor of POAF, focusing on the impact of nutritional status on surgical outcomes.

**Methods:**

This retrospective study included 321 patients who underwent CABG surgery between January 2010 and January 2024. TCBI was calculated using preoperative blood samples and compared between those who developed POAF and those who did not. Statistical analyses, including Cox regression and ROC analysis, were performed to assess the predictive value of TCBI for POAF. P<0.05 was considered statistically significant.

**Results:**

Patients who developed POAF had significantly lower TCBI (1790.8 ± 689, 3413.3±1232, p<0.001, respectively) levels compared to those without POAF. Also, age (p<0.001), the frequency of hypertension (p=0.009), CRP (p=0.03), and WBC (p=0.02) values were also significantly higher in patients who developed POAF.TCBI was identified as an independent predictor of POAF (OR: 0.998, 95% CI: 0.997-0.999, p<0.001), with a cut-off value of 1932.4 predicting POAF with 75% sensitivity and 78% specificity.

**Conclusion:**

The TCBI is a reliable indicator for predicting POAF in CABG patients. Preoperative identification of patients with low TCBI could lead to targeted interventions, reducing postoperative complications and improving outcomes. Optimizing nutritional status before surgery may mitigate the risk of POAF.

## Introduction

Atrial fibrillation (AF) is one of the common complications following cardiac surgery and remains the most prevalent type of arrhythmia after surgery. Postoperative atrial fibrillation (POAF) is defined as AF that develops within 1 to 5 days after cardiac surgery in patients without a prior diagnosis of AF. Fortunately, POAF often converts to sinus rhythm (SR) spontaneously within the first 24 hours.^1^ Studies have found that POAF develops in 25% to 50% of patients depending on the type of surgical procedure.^2^

The effect of nutritional status on cardiovascular surgery outcomes has long been recognized. Inadequate nutrition increases the risk of postoperative complications by improving processes such as inflammation and oxidative stress. Particularly in major surgical interventions like coronary artery bypass graft (CABG), good nutritional status in the preoperative period can accelerate postoperative recovery and reduce mortality.^3^ Simple and accessible nutritional indicators such as triglyceride-cholesterol-body weight Index (TCBI) are important tools for evaluating nutritional status in the preoperative period. Low TCBI levels are considered a sign of malnutrition and have been shown to be effective in predicting cardiovascular events in coronary artery disease patients.^4^ Therefore, optimizing nutritional support before major surgeries like CABG is crucial for reducing postoperative complications. Inadequate nutrition not only increases the risk of postoperative complications but also negatively affects overall quality of life and long-term survival. Indicators such as TCBI are valuable tools for predicting these risks and applying preventive nutritional interventions. In this context, accurately assessing patients' nutritional status before surgery is a critical step to prevent complications.

Despite advancements in surgical techniques and perioperative treatments, the incidence of POAF has not decreased over time. POAF continues to contribute to increased postoperative morbidity and mortality.^5^ Moreover, it remains a significant factor in healthcare costs.^6^ Notably, AF is observed more frequently in patients with POAF compared to those in postoperative SR.^7^

Several studies have demonstrated that POAF is associated with an increased incidence of short-term complications, particularly after coronary artery bypass graft (CABG) surgery.^8^ Furthermore, long-term studies have shown that POAF is linked with an increased risk of death and thromboembolic complications.^9^

In recent years, research has focused on biochemical and metabolic markers that may predict POAF, especially as preventive strategies could mitigate associated risks. One such marker is the TCBI, calculated as:


$$\text{TCBI}=\frac{\text{Triglyceride(mg/dL)}\times \text{Total cholesterol(mg/dL)}\times \text{Body weight(kg)}}{1,000}$$

Previous studies have shown that TCBI is a critical marker in patients with heart failure,^10^ coronary artery disease,^4^ critical illness,^11^ as well as in the general population and stroke patients.^12,13^ However, the relationship between TCBI and AF, particularly POAF, remains unexplored. Inadequate nutritional status not only increases the risk of postoperative complications but also negatively affects overall quality of life and long-term survival. Indicators such as TCBI are valuable tools for predicting these risks and applying preventive nutritional interventions. Therefore, accurately assessing patients' nutritional status before surgery is a critical step in preventing complications.

## Objective of the study

This study aims to investigate the relationship between TCBI and POAF by calculating TCBI from blood samples taken before CABG surgery. If a significant association is found, it may be possible to identify high-risk patients preoperatively and monitor them closely post-surgery. This could lead to reduced morbidity and mortality in patients prone to developing POAF, offering a new avenue for preventive care based on nutritional and metabolic risk factors.

## Methods

### Patient population and defining of TCBI

Our study was designed retrospectively. Between January 2010 and January 2024, 321 consecutive patients who underwent CABG surgery in our center were included in the study. The study design and flowchart are shown in the Central Illustration. The patient's files were evaluated retrospectively. The electrocardiograms (ECG) of the patients included in the study taken before CABG surgery were in SR. The patients were divided into two groups: those who developed postoperative AF and those who did not. Demographic characteristics, TCBI, and blood parameters of both groups were compared. The TCBI was calculated with this formula: ‘Triglycerides x Total cholesterol x Body weight/1,000'. The local ethics committee approved the present study. Our study was carried out in compliance with the ethical guidelines of the Declaration of Helsinki.

Criteria for inclusion in the study: these were defined as having no previous diagnosis of AF, undergoing CABG surgery, and having a documented sinus ECG before surgery.

Exclusion criteria: patients with electrolyte disorders, patients with severe heart valve diseases and chronic renal failure, patients with pacemakers, patients using antiarrhythmic drugs, patients with metabolic disorders, and patients without a preoperative SR ECG.

The definition of POAF was made as follows: in 5 days after cardiac surgery, arrhythmia lasting more than 10 minutes resolved spontaneously or was treated with electrical/medical cardioversion.^14^ Patients were closely monitored for arrhythmia throughout their hospital stay. An ECG was also taken when cardiac symptoms such as palpitations occurred. ECGs of the patients were recorded in 12 leads at 10 mm/mV and 25 mm/s settings.

### Statistical analysis

Histogram, q-q graph, and Shapiro-Wilk test were used to evaluate whether the data violated normality assumptions. A two-sample T-test was performed to compare continuous variables between groups. Chi-square analysis was used to evaluate the relationship between categorical variables. The continuous data were presented as mean ± standard deviation (SD) based on the data distribution. The categorical variables were expressed as the number (n) with a percentage (%). Cox regression analysis was used to determine the risk factors affecting POAF status. Variables that were found to be statistically significant as a result of Cox regression analysis were evaluated with univariate and multivariate Cox regression analysis. ROC (Receiver operating characteristic) analysis was performed to evaluate TCBI index and age in predicting POAF. The area under the curve and the cut-off value were calculated for each parameter value. Sensitivity and specificity were calculated to determine the diagnostic power of the scores. It was accepted that p-values should be <0.05 for the parameters to be statistically significant. Analysis of the data was performed in SPSS 22 statistical software.

## Results

Among the 321 patients included in the study, the number of patients who developed POAF after CABG was found to be 62. When the baseline clinical and demographic characteristics of both groups were compared, the age of the patients in the POAF group was higher than the other group. The number of hypertension was also higher in the POAF group than in the other group. CRP value was also found to be higher in the POAF group compared to the other group. WBC value was also found to be higher in the POAF group. Additionally, the TCBI was found to be lower in the POAF group than in the other group. There was no significant difference between the groups except in age, hypertension, CRP, WBC, and TCBI (Table 1).

**Table 1:** Comparison of baseline clinical, demographic, and laboratory characteristics between groups

	POAF (-) group (n = 259)	POAF (+) group (n = 62)	p Value
Age, years	61.96 ± 9.3	67.09 ± 6.5	<0.001
Female gender, n (%)	65 (25.1)	16 (25.8)	0.08
Length (cm)	170±7.1	169.3±7.3	0.72
Body weight (kg)	75.2±11.1	77.6±10.6	0.45
BMI (kg/m^2^)	26.80 ± 3.25	27.41 ± 3.36	0.67
Hypertension, n (%)	190 (73.3)	55 (88)	0.009
Diabetes Mellitus, n (%)	120 (46.3)	26 (41.9)	0.35
Hyperlipidemia, n (%)	230 (88.8)	55 (88.7)	0.81
Smoking, n (%)	121 (46.7)	22 (35.4)	0.08
Systolic BP (mmHg)	136.6 ± 12.8	139.1 ± 16.5	0.10
Diastolic BP (mmHg)	78.1 ± 8.3	79.3 ± 7.5	0.25
Creatinin (mg/dl)	0.9 ± 0.1	0.8 ± 0.15	0.17
Na (mmol/L)	138.7 ± 3.1	137.1 ± 2.2	0.90
K (mmol/L)	4.2 ± 0.2	4.3 ± 0.4	0.30
AST (U/L)	34 ±5.8	36±7.3	0.50
ALT (U/L)	31 ±6.6	36±9.2	0.51
Total cholesterol	234.3 ± 14.5	221.5 ± 15.1	0.60
LDL-C	146.5 ± 9.2	159.1 ± 9.6	0.12
CRP	62.6 ± 34.3	74 ± 38	0.03
WBC (10^3^/uL)	10.08 ± 3.5	11.7 ±4.7	0.02
Neutrophil (10^3^/uL)	8.6± 0.9	8.9 ±1.3	0.45
Hemoglobin (g/dL)	10.5 ± 1.2	11 ± 1.3	0.60
Platelet (10^3^/uL)	311.4 ± 37	369.1 ± 38	0.25
LVEF (%)	53.4 ± 7.6	51.5 ± 8.1	0.18
Left atrium diameter (mm)	44.2 ± 4.1	47.2 ± 5.2	0.09
LVSWT (mm)	10.3 ± 1.3	9.9 ± 2.1	0.51
PWT (mm)	9.2 ± 1.5	9.6 ± 1.8	0.89
LVEDD (mm)	48.9 ± 4.1	51.1 ± 3.9	0.13
LVESD (mm)	33.9 ± 3.4	33.4 ± 3.5	0.57
TCBI	3413.3±1232	1790.8 ± 689	<0.001

POAF: postoperative atrial fibrillation; BMI: body mass index; Na: sodium; K: potassium; AST: aspartate aminotransferase; ALT: alanine aminotransferase; LDL-C: low-density lipoprotein cholesterol; CRP: C-reactive protein; WBC: White blood cell; LVEF: left ventricular ejection fraction; LVSWT: left ventricular septal wall thickness; PWT: posterior wall thickness LVEDD: left ventricular end-diastolic diameter; LVESD: left ventricular end-systolic diameter; TCBI: triglyceride, total cholesterol, and body weight index.

Univariate and multivariate Cox regression analysis was performed to identify independent variables predicting POAF. According to multiple Cox regression analyses, age and TCBI were determined as strong independent predictors of POAF after CABG surgery (Table 2).

**Table 2:** The univariate and multivariate Cox regression analysis for predicting POAF

	Univariate		Multivariate	
	Odds Ratio (%95 CI)	p-value	Odds Ratio (%95 CI)	p-value
Age	1.060 (1.029 – 1.091)	<0.001	1.046 (1.014-1.080)	0.005
TCBI	0.997 (0.996 – 0.999)	<0.001	0.998 (0.997-0.999)	<0.001
Hypertension	2.599 (1.241 – 5.445)	0.011		
CRP	1.007 (1.001 – 1.014)	0.024		
WBC	1.091 (1.031 – 1.154)	0.003		

POAF: postoperative atrial fibrillation; TCBI: triglyceride, total cholesterol, and body weight index; CRP: C-reactive protein; WBC: white blood cell.

In ROC analysis, TCBI < 1932.4 was found to predict POAF with 75% sensitivity and 78% specificity (Figure 1).

**Figure 1:** ROC curve of Triglyceride-Cholesterol-Body Weight Index (TCBI).

## Discussion

This comprehensive study investigates the relationship between CABG surgery and POAF. The most significant finding of our study is that the TCBI was identified as an independent predictor of POAF.

Historically, AF was not considered a major complication following cardiac surgery. However, subsequent studies have demonstrated that POAF significantly impacts both mortality and morbidity.^15^ For instance, a study conducted with CABG patients revealed that those who developed POAF experienced longer durations on mechanical ventilation, as well as prolonged stays in the intensive care unit and hospital.^16^ Additionally, POAF has been associated with an increased long-term risk of mortality and stroke.^17^

In our study, it was observed that patients with low TCBI levels had a higher risk of POAF in the postoperative period. In the literature, the impact of nutritional status on surgical outcomes has been frequently emphasized, and it has been shown that malnutrition slows down the recovery process and increases the risk of complications.^18^ Given the role of inflammation in the development of AF, it is thought that poor nutritional status may exacerbate this process. Malnutrition has been shown to increase cardiovascular events and complications after cardiovascular surgery.^19^ TCBI not only predicts complications like POAF but also serves as a sensitive indicator of nutritional status. This finding suggests that patients with poor nutritional status may require more intensive nutritional support during the preoperative period. Furthermore, our study confirms that compared to other nutritional indicators, TCBI more specifically reflects malnutrition and the risk of complications. A better understanding of the relationship between nutritional status and POAF could contribute to the development of personalized nutritional approaches in these patients, potentially reducing postoperative complications and improving overall health outcomes. Improving nutritional status could also alleviate the burden on healthcare systems by reducing postoperative complications in the long term.

Given the potential for POAF to lead to serious postoperative complications, identifying patients at high risk for AF has become a critical goal. This understanding has driven researchers to conduct more comprehensive studies on the risk factors associated with POAF. It is well known that poor nutritional status triggers inflammation, which may pave the way for complications such as AF after surgery. Nutritional indices such as TCBI are considered important tools in predicting these complications, and preventive nutritional interventions may be applied, particularly in high-risk patients.

Several studies have highlighted factors that may contribute to the development of POAF. Hypoxemia has been identified as a significant risk factor for POAF,^20^ while other research has demonstrated a correlation between various surgical techniques and the incidence of POAF.^21^ Risk factors such as advanced age, increased left atrial (LA) diameter, reduced ejection fraction (EF), chronic obstructive pulmonary disease (COPD), hypertension, myocardial infarction, and diabetes have all been associated with POAF development, as supported by a meta-analysis involving 36,834 patients.^22^

The value of TCBI as a significant predictor of outcomes in various diseases, particularly cardiovascular conditions, has been increasingly recognized. Research has shown that low TCBI levels are strongly associated with all-cause mortality and cardiovascular mortality in patients with coronary artery disease.^4^ Furthermore, TCBI is recommended for assessing the nutritional status of cardiovascular patients, as it has proven to be a strong predictor of poor prognosis and mortality in both cardiovascular patients and the general population.^4^ In our study, TCBI was found to be a significant predictor of postoperative AF in patients undergoing CABG surgery.

Inflammatory markers also play a significant role in the development of AF. Many studies have shown that patients with AF tend to have higher levels of inflammatory markers compared to those in SR.^23^ Additionally, C-reactive protein (CRP) levels have been shown to predict the development of new-onset AF.^24^ In our study, elevated white blood cell (WBC) counts and CRP levels in the group that developed POAF suggest that an inflammatory mechanism may be contributing to the onset of AF.

Age has consistently been identified as an independent risk factor for the development of AF. One study estimated that the prevalence of AF in elderly patients in the European Union would more than double after the age of 50.^25^ Our findings align with these observations, as we identified advanced age as one of the most significant risk factors for the development of POAF.

### Limitations

The most important limitation of the study is that it is retrospective. Lack of sufficient knowledge about the surgical techniques applied is also an important limitation. We also do not have data on the anesthetic drugs given to patients before surgery. The anesthetic agents given may also have changed this parameter. Multicenter, prospective, randomized controlled studies are needed to understand better whether this parameter is predictive of POAF.

## Conclusions

TCBI can predict the development of POAF in patients undergoing CABG. With the help of this parameter, patients who need to receive prophylactic treatment before CABG can be determined, thus reducing mortality and morbidity.
